# The association between let-7, RAS and HIF-1α in Ewing Sarcoma tumor growth

**DOI:** 10.18632/oncotarget.5616

**Published:** 2015-09-10

**Authors:** Michal Hameiri-Grossman, Adi Porat-Klein, Isaac Yaniv, Shifra Ash, Ian J. Cohen, Yona Kodman, Ronit Haklai, Galit Elad-Sfadia, Yoel Kloog, Elena Chepurko, Meora Feinmesser, Josephine Issakov, Osnat Sher, Drorit Luria, Yehuda Kollender, Avraham Weizman, Smadar Avigad

**Affiliations:** ^1^ Molecular Oncology, Felsenstein Medical Research Center, Rabin Medical Center, Petah Tikva, Israel; ^2^ Pediatric Hematology Oncology, Schneider Children's Medical Center of Israel, Petah Tikva, Israel; ^3^ Department of Neurobiochemistry, The George S. Wise Faculty of Life Sciences, Tel Aviv University, Tel Aviv, Israel; ^4^ Pathology Department, Rabin Medical Center, Petah Tikva, Israel; ^5^ Pathology Department, Sourasky Medical Center, Tel Aviv, Israel; ^6^ Unit of Orthopedic Oncology, Sourasky Medical Center, Tel Aviv, Israel; ^7^ Sackler Faculty of Medicine, Tel Aviv University, Tel Aviv, Israel

**Keywords:** microRNA, let-7, RAS, Ewing sarcoma, HIF-1α

## Abstract

Ewing Sarcoma (ES) is the second most common primary malignant bone tumor in children and adolescents. microRNAs (miRNAs) are involved in cancer as tumor suppressors or oncogenes. We studied the involvement of miRNAs located on chromosomes 11q and 22q that participate in the most common translocation in ES. Of these, we focused on 3 that belong to the *let-7* family.

We studied the expression levels of *let-7a*, and *let-7b* and detected a significant correlation between low expression of *let-7b* and increased risk of relapse. *let-7* is known to be a negative regulator of the RAS oncogene. Indeed, we detected an inverse association between the expression of *let-7* and RAS protein levels and its downstream target p-ERK, following transfection of *let-7* mimics and inhibitors. Furthermore, we identified *let-7* as a negative regulator of HIF-1α and EWS-FLI-1. Moreover, we were able to show that HIF-1α directly binds to the EWS-FLI-1 promoter. Salirasib treatment *in-vitro* resulted in the reduction of cell viability, migration ability, and in the decrease of cells in S-phase. A significant reduction in tumor burden and in the expression levels of both HIF-1α and EWS-FLI-1 proteins were observed in mice after treatment.

Our results support the hypothesis that *let-7* is a tumor suppressor that negatively regulates RAS, also in ES, and that HIF-1α may contribute to the aggressive metastatic behavior of ES. Moreover, the reduction in the tumor burden in a mouse model of ES following Salirasib treatment, suggests therapeutic potential for this RAS inhibitor in ES.

## INTRODUCTION

Ewing Sarcoma (ES) is the second most common primary malignant bone tumor in children and adolescents [1]. ES is characterized by specific translocations fusing the EWS gene with different members of the ETS transcription family, the most frequent is the EWS-FLI-1 fusion [2, 3]. ES is an aggressive disease and despite advances in therapy, up to 40% of the patients will eventually relapse [4].

microRNAs (miRNAs) are small non-coding RNAs that play a role in post-transcriptional regulation. miRNAs have been shown to be involved in many cellular processes, including in cancer as tumor suppressors or oncogenes [5]. One of the most studied miRNA is *let-7*. The human *let-7* family consists of 13 genes, is highly conserved and has been found to be downregulated in several malignancies such as lung cancer, colon, breast, and acute lymphoblastic leukemia [6]. One of the validated targets of *let-7* is the RAS oncogene [7].

One of the downstream targets of the RAS pathway is HIF-1α, which plays a key role in cellular response to hypoxia [8]. It is overexpressed in many types of cancer and its level of expression correlates with metastatic disease and mortality [9]. Positive HIF-1α expression was identified in a large fraction of primary ES tumors [10] [11]. Interestingly, Aryee et al. have shown that EWS-FLI-1 is upregulated by HIF-1α and De Vito et al. have shown that EWS-FLI-1 is a direct target of *let-7* [11] [12]. HIF-1α transactivates the expression of a multitude of target genes, which are implicated in events such as angiogenesis, cell survival, migration and metastasis [13]. Under normoxic conditions, HIF-1α is targeted for ubiquitin-mediated degradation by the proteasome, and has a half-life of less than five minutes. HIF-1α can also be regulated through oxygen-independent mechanisms such as phosphoinositide 3-kinase (PI3K)-Akt-mTOR and RAS/MAPK pathways [8].

ERK was found to be constitutively activated in cells expressing EWS-FLI-1. Inhibition by MEK1 or a dominant negative *RAS* mutant impaired EWS-FLI-1 dependent transformation [14].

The primary aim of our work was to identify mechanisms leading to the aggressiveness of ES.

We found the *let-7* family to be downregulated in ES, leading to the activation of the RAS pathway and HIF-1α. Moreover, we showed that EWS-FLI-1 is up-regulated through HIF-1α that directly binds to the promoter of EWS. Furthermore, we demonstrated that Salirasib, a RAS inhibitor, significantly reduced HIF-1α and EWS-FLI-1 proteins and tumor growth in an ES mouse model. Our findings clarified the role of RAS and HIF-1α in ES and identified a potential active agent against ES tumors, to be explored in clinical studies.

## RESULTS

We were interested in miRNAs located on chromosomes 11 and 22 that are involved in the most prevalent translocation in ES resulting in the chimeric transcript EWS-FLI-1. Using miRBase, 10 miRNAs were identified on chromosome 11 and 5 on chromosome 22. Interestingly, 3 out of the miRNAs identified belong to the *let-7* family: *let-7a-2* (chromosome 11), *let-7a* and *let-7b* (chromosome 22). We decided to focus on *let-7* family for further analysis.

### *let-7* expression levels are predictive of outcome

**Figure 1:**
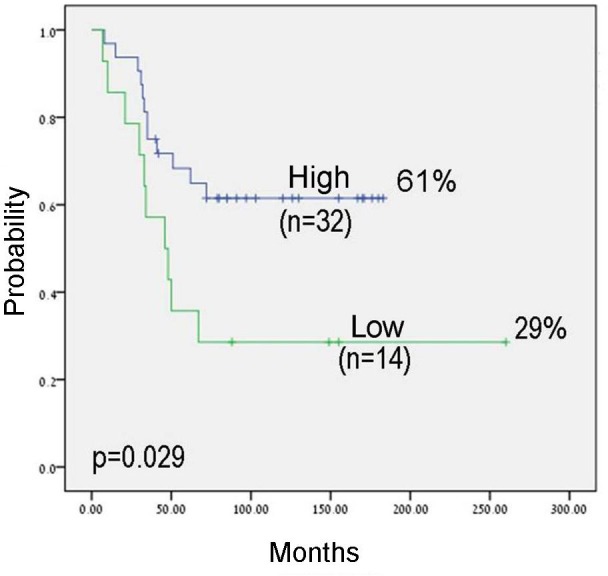
Kaplan-Meier analysis for progression free survival (PFS) by *let-7b* expression levels in patients with localized disease The cut-off for expression levels was determined by the first quartile: high expression: equal and above the quartile; low expression: under the quartile.

We evaluated the expression levels of *let-7a* and *let-7b* by RQ-PCR in 57 primary ES tumors. We used the expression levels of the first quartile as a cut-off for the definition of high and low/negative expression levels. No significant correlation was detected between expression levels and clinical parameters. However, in the group of patients with localized disease, a significant correlation with outcome was observed. Progression free survival (PFS) at ten years for patients with high *let-7b* expression was 62% versus 29% for those with low *let-7b* expression level (*p* = 0.029; Figure 1). Multivariate Cox regression analysis including age, primary site, metastasis at diagnosis and expression of *let-7a* and *let-7b* identified expression of *let-7b* as an independent prognostic factor in ES, with a 4.4 fold increased risk of relapse (*p* = 0.021, 95% confidence interval 1.2-15.5).

### Activation of the RAS pathway

**Figure 2:**
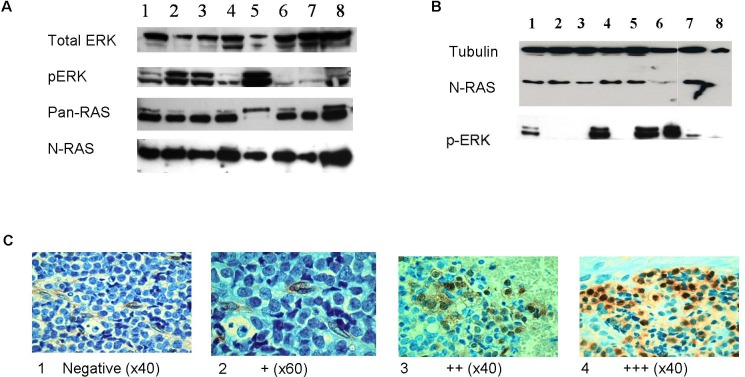
RAS signaling pathway is activated in ES **A.** Western blot analysis of total ERK, p-ERK, pan-RAS and N-RAS proteins in Ewing cell lines (1-SK-N-MC, 2-SK-N-MC-II, 3-SK-ES1, 4-RD-ES1, 5-MHH-ES1, 6-EW-2, 7- TCL-9, 8-5838). RAS and p-ERK proteins are expressed in all cell lines. **B.** Representative Western blot analysis of N-RAS and p-ERK in 8 ES primary tumors. N-RAS was detected in 7 tumors (no. 1-7) and p-ERK in 5 tumors (no. 1,3,5,6,7). **C.** Representative pictures of immunohistochemistry staining of anti p-ERK (p44/42) in ES paraffin-embedded tumors at diagnosis. Positive p-ERK staining is in brown and was scored as: 1:Negative; 2:weak (+); 3:moderate (++); 4:strong (+++).

We measured the protein levels of RAS and its downstream target p-ERK in ES cell lines and primary tumors. All eight cell lines studied expressed RAS protein and p-ERK (Figure 2A), indicating that the RAS signaling pathway is activated. RAS protein levels were measured by Western blot in only 13 tumors, since we did not have sufficient fresh tumor material from all patients. All these 13 tumors expressed RAS and 8 (62%) of them expressed p-ERK (Figure 2B). Additional 45 paraffin-embedded primary tumors were analyzed for p-ERK protein levels by immunohistochemistry staining. Positive p-ERK immuno-staining was detected in 31 out of the 45 (69%) tumors analyzed; of them, 17 (38%) were defined as strong (+++) expression, 12 (27%) as moderate (++) expression, 2 (4%) as weak (+) expression and 14 (31%) were negative (Figure 2C).

No significant correlation was identified between p-ERK protein expression levels and clinical parameters or outcome. In 33 tumor samples, the expression results of both *let-7* and p-ERK protein were available and a significant inverse correlation was identified between *let-7a* and p-ERK expression levels (*p* = 0.037).

Since the RAS signaling pathway can be activated by mutations of the *RAS* genes, we performed mutation analysis of the hot spots in all 3 *RAS* genes in 27 available DNA samples from primary tumors. None of the tumors harbored a mutation.

**Figure 3:**
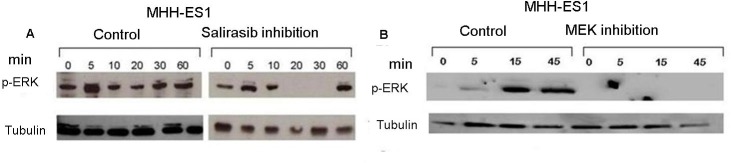
Inhibition of RAS signaling pathway by Salirasib and U0126 Cells were serum starved for 12 hrs and then treated with Salirasib for 1 hr and U0126 for 5 hrs. **A.** p-ERK protein levels were measured following serum reactivation for 5 - 60 minutes post-Salirasib treatment **A.** and for 5-45 minutes post-U0126 treatment **B.**

Validation of the activated RAS signaling pathway was performed by using 2 drugs that inhibit two participants of this pathway: Salirasib targeting RAS and U0126 targeting MEK1. Inhibition by both Salirasib and U0126 resulted in the reduction of p-ERK protein expression after serum activation of 20 or 45 minutes, respectively (Figures 3A and 3B).

### Expression of HIF-1α protein in normoxia

**Figure 4:**
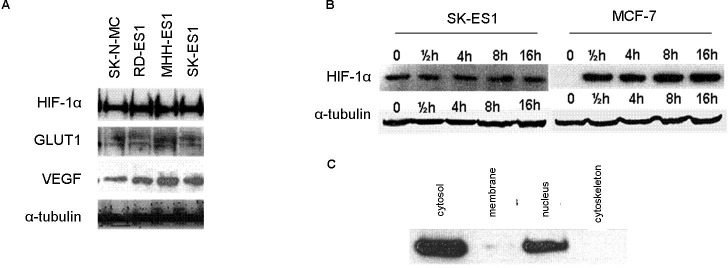
Expression of HIF-1α and its targets in ES **A.** Western blot analysis of HIF-1α, GLUT1 and VEGF protein expression levels in ES cell-lines under normoxia. The proteins are expressed in all 4 cell lines. **B.** HIF-1α protein expression levels in SK-ES1 and MCF7 cell lines under induced hypoxia (1% O_2_) at different time points. In ES, HIF-1α is expressed during normoxia (0) and hypoxia, in contrast to a breast cancer line (MCF7) in which it is expressed only during hypoxia. **C.** HIF-1α protein is identified in the cytosolic and nuclear fractions following cell fractionation assay.

The protein expression levels of HIF-1α were measured in ES cell lines under normoxic conditions (21% oxygen) (Figure 4A). HIF-1α protein was expressed in all cell lines studied. We measured the protein expression levels of 2 known target genes of HIF-1α, VEGF and GLUT-1 and detected expression in all cell lines that were studied (Figure 4A).

Since HIF-1α is usually overexpressed during hypoxia, we measured the protein levels of HIF-1α also under hypoxic conditions (1% O_2_) using a Hypoxia Chamber (Billups-Rothenberg, San Diego, CA, USA). The cells were exposed to hypoxia for different durations and analyzed at the following time points: 0.5 hr, 4 hrs, 8 hrs and 16 hrs (Figure 4B). We detected no difference in HIF-1α protein expression levels during hypoxia for any of the time points compared to the level in normoxia. HIF-1α protein expression levels during hypoxia were similar to the basal condition without hypoxia. This is in contrast to the breast cancer MCF7 cell line in which HIF-1α is expressed only under hypoxia (Figure 4B). We examined the cellular localization of the HIF-1α protein during normoxia. HIF-1α protein was detected both in the nucleus and in the cytoplasm under normoxic conditions (Figure 4C).

### HIF-1α directly binds to EWS-FLI-1

**Figure 5:**
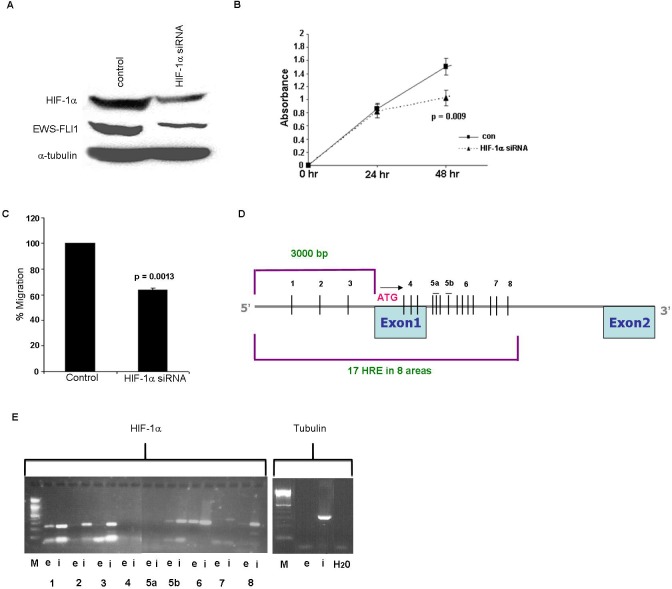
Silencing of HIF-1α gene and HIF-1α binding to EWS **A.** Western blot analysis of HIF-1 and EWS-FLI-1 proteins following silencing of HIF-1α in SK-ES1 cell line, resulted in a decrease in HIF-1α and in EWS-FLI-1 protein levels. **B.** Silencing of HIF-1α led to a decrease in cell growth following 48 hrs using XTT proliferation analysis. **C.** The migration ability of the cells was measured by Cell Migration assay following silencing of HIF-1α. **D.** An illustration of 17 HREs 3000bp upstream the start codon and part of the intron of EWS. **E.** Proteins cross-linked to chromatin from SK-ES1 cells were precipitated with specific antibody directed against HIF-1α and the associated DNA sequences were amplified with PCR primers that amplify the 8 regions. PCR products from input chromatin before- (i- input) and after immunoprecipitation (IP)- (e-elution) are shown.

In order to evaluate if HIF-1α regulates EWS-FLI-1, ES cell lines were transfected with HIF-1α siRNA. The silencing resulted in a significant decrease of 66% (*p* = 0.004) in HIF-1α protein (Figure 5A). Following silencing, we observed a significant downregulation of 61% (*p* = 0.003) of EWS-FLI-1 protein levels (Figure 5A). The biological effect of HIF-1α silencing was assessed by cell proliferation and migration assays. Following silencing for 48 hrs, a reduction of 32% (*p* = 0.009) in the cell proliferation rate was identified (Figure 5B). This was followed by a significant 36% decrease (*p* = 0.0013) in the ability of the cells to migrate (Figure 5C).

Our results suggesting that HIF-1α regulates EWS-FLI-1 led us to examine whether HIF-1α directly binds the chimeric transcript EWS-FLI-1. The EWS promoter region was searched for Hypoxia Response Element (HRE) consensus sequence comprised of CGTC, to which HIF-1α binds and upregulates the transcription of target genes. Looking over 3000 bp upstream to the transcription initiation site and into the first intron of EWS, we detected 17 HREs: some of them were isolated and others were grouped in clusters of three or four CGTG sequences (Figures 5D, 5E). We divided the region containing the 17 HREs into 8 equal sub-regions and designed primers for each sub-region for the ChIP assay. Sub-regions 1, 2, 3, 5b and 8 contained 1 HRE, while sub-regions 4 and 5a contained 3 HREs, sub-region 6 contained 4 HREs and sub-region 7 contained 2 HREs (Figure 5D). Immunoprecipitated DNA product was detected in sub-regions 1, 2, 3, 5b, 6 and 8 (Figure 5E). Tubulin was used as a control. These results confirm that HIF-1α directly binds to EWS in at least 6 of the 8 HREs. Area 6 contains 4 HREs that could not be individually identified.

### *let-7* regulates the expression of RAS, p-ERK, HIF-1α and EWS-FLI-1

**Figure 6:**
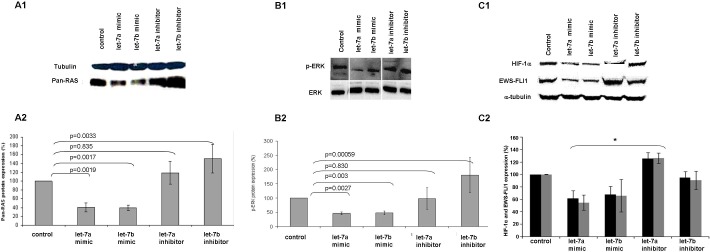
*let-7* regulates the expression of RAS, p-ERK, HIF-1α and EWS-FLI-1 Western blot analysis of pan-RAS, p-ERK, HIF-1α and EWS-FLI-1 proteins (A1, B1 and C1, respectively) following transfection of *let-7* mimics and inhibitors. Graphical representation following densitometry calculation (A2, B2 and C2) showing protein expression change from control expressed in percentage. *T*-test was used. * Significant result.

Synthetically, we overexpressed and silenced the levels of *let-7a* and *let-7b* in ES cells following transfection with mimics or inhibitors and measured the levels of RAS, p-ERK, HIF-1α and EWS-FLI-1 proteins (Figure 6). The protein expression levels were compared to a control cell line transfected only with a transfection reagent or with a scramble sequence.

RAS protein levels were significantly reduced following transfection with *let-7a* and *let-7b* mimics (59% and 61%, *p* = 0.0019 and *p* = 0.0017, respectively) (Figure 6A1 and 6A2).

Similarly, p-ERK levels were significantly reduced by 55% and 52% (*p* = 0.027 and *p* = 0.003, respectively) (Figure 6B1 and 6B2). A significant reduction was also evident in HIF-1α and EWS-FLI-1 levels following transfection with *let-7a* (38% and 45%, *p* = 0.006 and 0.0028, respectively) and *let-7b* mimics (31% and 401%, *p* = 0.006 and *p* = 0.01, respectively) (Figure 6C1 and 6C2).

This phenomenon was reversed following transfection with *let-7a* and *let-7b* inhibitors. We identified an increase of RAS and p-ERK levels (51% and 80%, *p* = 0.0033 and *p* = 0.00059, respectively) following the silencing of *let-7b* (Figures 6A and 6B) and an increase of HIF-1α and EWS-FLI-1 (26% and 27%, *p* = 0.01 and *p* = 0.0052, respectively) following silencing of *let-7a* (Figure 6C).

These results suggest that *let-7* regulates EWS-FLI-1 and HIF-1α, in addition to RAS and its downstream target.

### Salirasib treatment *in-vitro*

**Figure 7:**
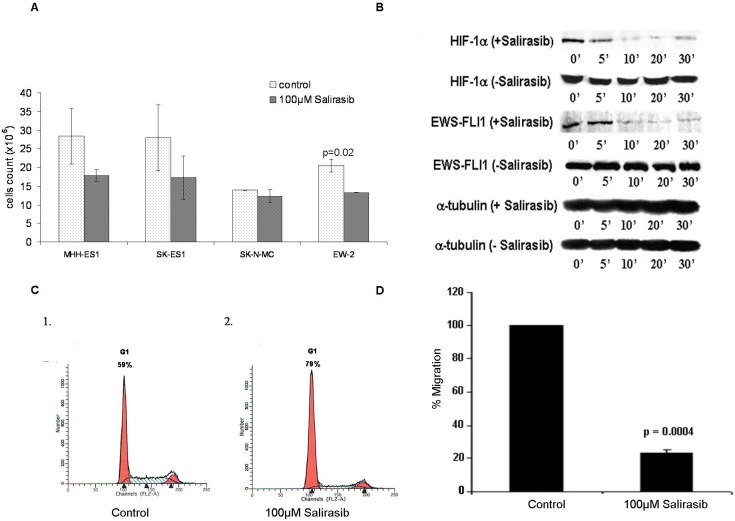
Effect of Salirasib treatment *in-vitro* **A.** Cells were treated with 100μlM Salirasib for 48 hours and the amount of viable cells were counted versus control cells in 4 ES cells lines. **B.** Western blot analysis of HIF-1α and EWS-FLI-1 proteins following Salirasib treatment. Cells were serum starved for 12 hrs and then treated by Salirasib for 1 hr and followed by serum reactivation for 5 - 30 minutes. A significant decrease in HIF-1α and EWS-FLI-1 protein levels is evident after 10 minutes. **C.** Cell cycle analysis by FACS was performed in cells treated with Salirasib and control. An increase in G1 phase is evident following Salirasib treatment. **D.** The migration ability of the cells was measured by Cell Migration assay following treatment with Salirasib.

Since our results imply that the RAS pathway is activated in ES, we tested the effect of the RAS inhibitor, Salirasib on cell count of MHH-ES1, SK-ES1, SK-N-MC and EW-2 cell lines (Figure 7A). A reduction in cell count was evident in all cell lines after 48 hours of treatment.

Following *in-vitro* Salirasib treatment for an hour, a significant reduction of 84% (*p* = 0.003) and 80% (*p* = 0.006) in HIF-1α and in EWS-FLI-1 protein levels, respectively, was detected 10 minutes following serum activation (Figure 7B) and a reduction of 100% in p-ERK protein levels 20 minutes following serum activation (Figure 3A).

Furthermore, an increase in the proportion of cells in G1-phase (from 59% to 79%) together with a decrease in S-phase (from 30% to 13%) was detected, indicating a G1 arrest following treatment (Figure 7C).

A decrease of 80% in the migration ability of ES cells was evident following treatment (*p* = 0.0004; Figure 7D).

### Salirasib treatment *in-vivo*

**Figure 8:**
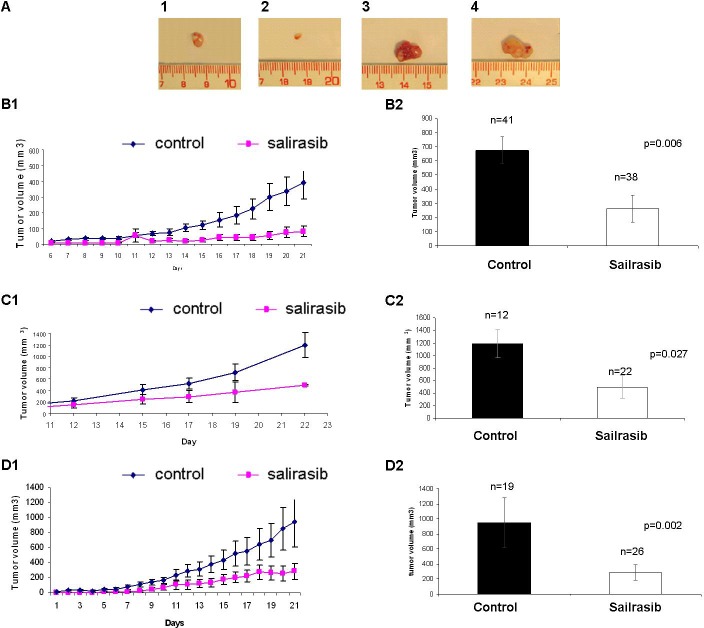
Effect of Salirasib treatment *in vivo* **A.** Following 18 days of Salirasib treatment, the mice (injected SK-ES1 cells) were sacrificed and the tumors were excised. Tumors from treated mice are shown in 1 and 2 and from control mice 3 and 4. **B.** Mice injected with SK-ES1 cells. B1: Representative graph of tumor volume measured daily from the beginning of treatment. Control (*n* = 41, tumor volume on day 21: 397±92mm^3^), Salirasib treated (*n* = 38, tumor volume on day 21: 82±95mm^3^). B2. Tumor volume of the tumors excised after the mice were sacrificed in the treated and control mice. **C.** Mice injected with A673 cells. C1: Representative graph of tumor volume measured daily from the beginning of treatment Control (*n* = 12, tumor volume on day 22: 1189±219mm^3^), Salirasib treated (*n* = 22, tumor volume on day 22: 493±180mm^3^). C2: Tumor volume of the tumors excised after the mice were sacrificed in the treated and control mice. **D.** Representative graph of tumor volume measured daily from the beginning of treatment in the microscopic disease setting. Control (*n* = 19, tumor volume on day 21: 955±204mm^3^), Salirasib treated (*n* = 26, tumor volume on day 22: 321.±79mm^3^). D2: Tumor volume of the tumors excised after the mice were sacrificed in the treated and control mice.

The effect of Salirasib was tested *in-vivo* in an ES xenograft mouse model generated by 2 different ES cell lines (SK-ES1 and A673) in independent experiments on a group of 113 mice: 60 were treated and 53 served as control. The mice were treated for 16-22 days from the day the tumors were clinically evident in all mice. The volume of the tumors was measured daily until the mice were sacrificed. Following the excision of the tumors at the end of the experiment, a difference in tumor size between treated and non-treated mice was evident (Figure 8A). On the day the mice injected with SK-ES1 were sacrificed, the mean volume of the tumors in the treated group was 82±45mm^3^, significantly lower than in the control group which was 392±43.45mm^3^ (*p* = 0.011; Figure 8B1 and Figure 8B2). In the mice injected with A673, the mean volume of the tumors in the treated group was 493±180mm^3^, significantly lower than in the control group which was 1189±219mm3 (*p* = 0.021; Figure 8C1 and Figure 8C2).

**Figure 9:**
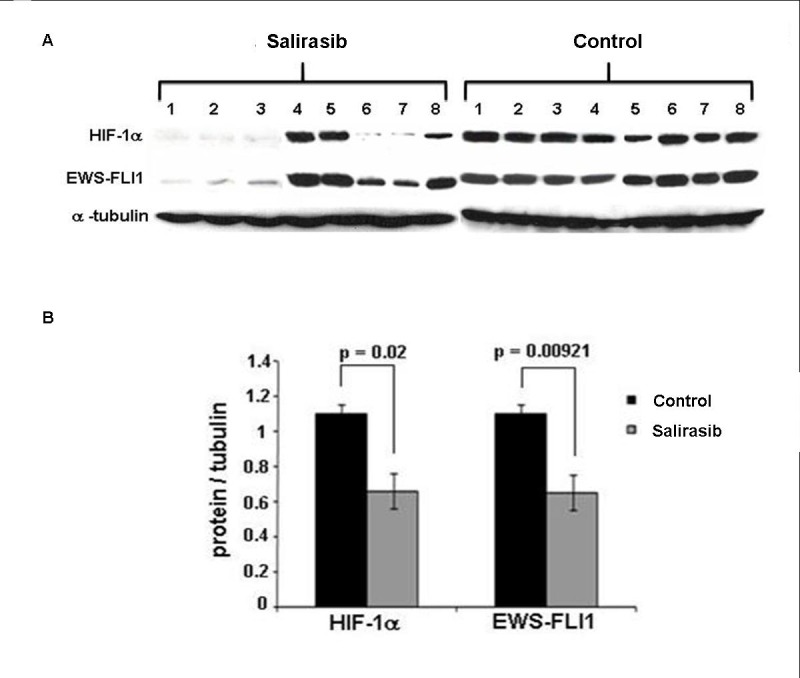
HIF-1α and EWS-FLI-1 levels following Salirasib treatment *in vivo* **A.** Following treatment with Salirasib for 21 days, the mice were sacrificed and tumors were excised. HIF-1α and EWS-FLI-1 proteins were measured by Western Blot. **B.** Densitometric illustration of the Western blot. A significant reduction of HIF-1α and EWS-FLI-1 proteins is evident following Salirasib treatment.

The effect of Salirasib treatment on microscopic disease was studied in 3 independent experiments including 45 mice (19 control and 26 treated mice) in which treatment was initiated simultaneously with the injection of the SK-ES1 cells. Salirasib treatment significantly inhibited tumor growth and the tumor mean volume at the end of the experiment observed was 321±79.15mm^3^ in the treated mice versus 955±203.95mm^3^ in the control group (*p* = 0.002, Figure 8D1 and 8D2). Of note, 2 mice in the treated group did not develop a tumor until the end of the experiment. In addition, following Salirasib treatment, the level of HIF-1α and EWS-FLI-1 protein levels were measured in 8 treated mice and compared to 8 control mice. A significant reduction of 58% and 67% in HIF-1 (*p* = 0.02) and EWS-FLI-1 (*p* = 0.00921) protein levels, respectively, was noted (Figure 9).

## DISCUSSION

Our aim in this work was to reveal potential pathways that contribute to the aggressiveness of ES, through microRNA analysis. miRNAs have been shown to be located at fragile sites and at cancer associated regions [19]. We were interested in miRNAs located on the chromosomes most frequently translocated in ES tumors, chromosomes 11q and 22q that result in the chimeric transcript EWS-FLI-1. Of the miRNAs located at the site of interest, 3 are members of the known *let-7* family. We showed that *let-7* is downregulated in ES and is significantly associated with poor outcome and an increase in relapse rates. Our results are consistent with a recent study providing evidence that *let-7* is downregulated and functions as a tumor suppressor gene in ES [20]. Previous studies have shown similar results for adult malignancies such as lung, prostate and glioblastoma [21-23]. Additional confirmation of the involvement of the *let-7* family in ES came from the miRNA microarray analysis we performed on 30 primary tumors using the miRXplore microarrays (Miltenyi Biotec, Bergisch Gladbach, Germany). We detected the downregulation of all of the *let-7* members in all ES tumors analyzed. This is supported by De Vito et al.'s findings [12] demonstrating the repression of the entire *let-7* tumor suppressor family in ES cell lines.

Studies have shown that *let-7* directly binds the RAS oncogene, resulting in negative regulation of RAS protein [7, 24-26]. Indeed, we were able to show that the RAS signaling pathway was activated in ES tumors and cell lines by the expression of p-ERK. We were able to confirm the RAS signaling pathway by using 2 inhibitors targeting RAS and the downstream kinase, MEK1. Both, RAS and MEK1 inhibitors, completely abolished p-ERK expression. Additional confirmation of the activated RAS signaling pathway came from the study by Silvany et al. who demonstrated constitutive ERK activation in ES cell lines, which was proportional to the level of EWS-FLI-1 expression [14].

The RAS inhibitor, Salirasib, is a RAS farnesyl cysteine mimetic that selectively disrupts the association of active RAS proteins and the plasma membrane. Salirasib blocks the functions of RAS proteins by interfering with their trafficking to and from cellular membranes, as well as with their proper localizations in different cellular location and microdomain [17], all of which are required for RAS signaling and biological activities. U0126 is a MEK inhibitor chemically synthesized organic compound that inhibits selectively the kinase activity of MAP kinase. In an attempt to explore whether oncogenic mutations in the RAS genes also contribute to the activation of the RAS pathway, we directly sequenced all the hot spot mutations in the 3 RAS genes but no mutation was identified. This was not surprising since RAS mutations are extremely rare in ES [27].

Since HIF-1α is stabilized and regulated by RAS and has been identified in ES primary tumors [10] [11], we explored the expression levels of HIF-1α protein and its correlation with EWS-FLI-1.

HIF-1α is a transcription factor, which is expressed in many cell types in response to oxygen deprivation and it's overexpression correlates with poor patient outcome [28-30].

Little is known about the involvement of HIF-1α in pediatric tumors. Overexpression of HIF-1α protein was detected in 79% of osteosarcoma tumors. Those patients expressing high levels of HIF-1α protein showed significantly lower overall and disease free survival [30]. In an additional study on osteosarcoma tumors, a significant correlation was observed between increased HIF-1α expression and metastases in patients [31]. Neuroblastoma cell lines were shown to stabilize HIF-1α in hypoxia and to up-regulate hypoxia-responsive genes, including VEGF [32].

We detected expression of HIF-1α and its targets, GLUT1 and VEGF proteins in ES cell lines under normoxic conditions. Similarly, expression of HIF-1α protein was found in pancreatic cancer cells and cholangiocarcinoma cells under normoxia [33]. In osteoblast and osteosarcoma cell lines, low HIF-1α expression levels were identified under normoxia which increased when the cells were moved to hypoxic conditions [34]. In ES, we detected similar levels of HIF-1α protein expression in normoxia and hypoxia. In skeletal muscle, HIF-1α was also found to be highly expressed during normoxia, as well as in hypoxia [35].

Our results are in contrast to those published by Aryee et al., in which HIF-1α protein expression was detectable under hypoxic but not normoxic conditions in ES cell lines [11]. However, Knowles et al., identified HIF-1α protein expression during normoxia in the same 3 cell lines that we studied [10].

Under normoxic conditions, HIF-1α is hydroxylated and targeted by the von Hippel-Lindau protein (pVHL) E3 ubiquitin ligase complex, leading to proteasomal degradation [36]. During hypoxia, cytoplasmic HIF-1α is imported into the nuclei, generating a DNA binding complex together with HIF-1β [35, 36]. Immunofluorescence analysis revealed that under normoxic conditions, significant amounts of HIF-1α can be found exclusively in the cytoplasm [35]. We detected HIF-1α protein both in the nucleus and in the cytoplasm in ES under normoxia, supporting the identified activation of HIF-1α in ES. This is consistent with Knowles et al., who demonstrated that HIF-1α was predominantly localized in the nucleus of Ewing's tumor cells [10]. HIF-1α expression can be regulated by various oncogenic pathways. For instance, insulin, insulin-like growth factor, SCF/c-kit and mutant BRAF can induce the expression of HIF-1α protein in normoxia via mitogen-activated protein kinase (MAPK) and PI3K/Akt pathways [37-40]. In addition, transformation by H-RAS has also been shown to enhance the levels of HIF-1α protein expression under normoxia [41]. We suggest that RAS signaling pathway stabilizes HIF-1α in ES.

We showed that HIF-1α directly binds and regulates EWS-FLI-1 protein. This is consistent with the study by Aryee et al. [11] who suggested that HIF-1α regulates EWS-FLI-1. Both studies add EWS-FLI-1 to the growing list of HIF-1α target genes.

The phenotypic consequence of HIF-1α silencing was a significant decrease in cell growth and migration ability of the cells. Our results are similar to those by Aryee et al. confirming that hypoxia stimulated the invasiveness and soft agar colony formation of ESFT cells *in-vitro* [11]. Their findings together with ours suggest that HIF-1α may contribute to the aggressive metastatic behavior of ES.

We hereby describe that *let-7* expression inversely correlated with the levels of RAS, p-ERK, HIF-1α and EWS-FLI-1 proteins. We suggest that the expression of EWS-FLI-1 and p-ERK proteins are regulated by *let-7*. This is of extreme importance for a possible therapeutic use of *let-7* in ES, since the EWS-FLI-1 protein is known to be a pivotal oncogenic event in ES. De Vito et al. [12] demonstrated that *let-7* is a direct EWS-FLI-1 target resulting in reduced *let-7a* expression which is implicated in ESFT development through HMGA2 regulation. Interestingly, their miRNA based therapy using synthetic *let-7* supports our findings suggesting that increased levels of *let-7* result in a reduction of HIF-1α and EWS-FLI-1, contributing to the reduction of tumor growth *in-vivo*.

**Figure 10:**
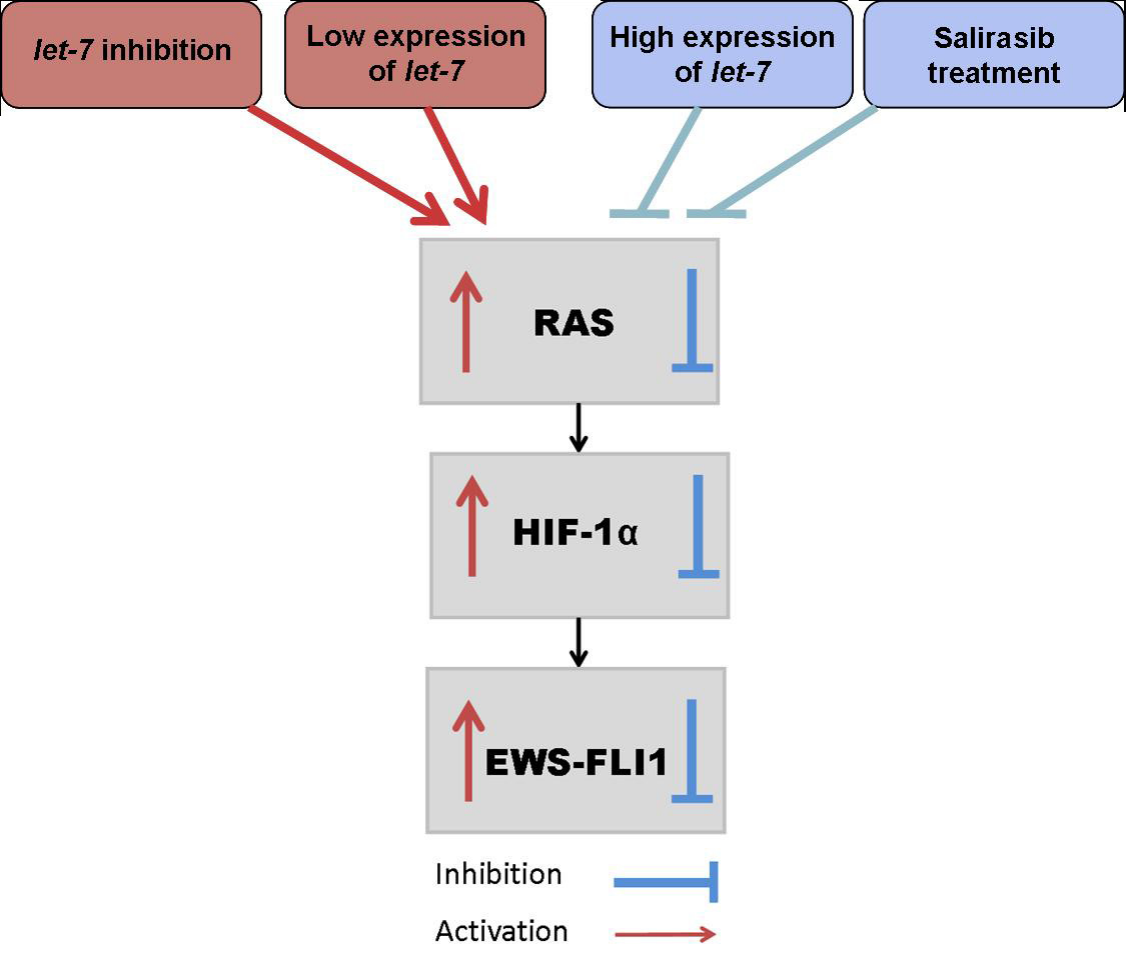
let-7/RAS/HIF-1α /EWS-FLI-1 circuit in ES Downregulation of let-7 results in the activation of the RAS signaling pathway leading to the activation of HIF-1α and this leads to an increase in EWS-FLI-1. Salirasib treatment or transfection with *let-7* mimic inhibits RAS signaling pathway, HIF-1α and EWS-FLI-1.

Based on the results of both studies we hypothesize the possible existence of a feedback loop in which EWS-FLI-1 binds *let-7* resulting in downregulation of *let-7*, which in turn upregulates EWS-FLI-1 (Figure 10).

Next we evaluated the clinical use of Salirasib in ES. Salirasib has been shown to specifically inhibit active RAS by inhibiting RAS-driven RAF/MEK/ERK pathway and arresting cell growth. These results were obtained for several types of cancer such as melanoma, pancreatic carcinoma, glioblastoma, neuroblastoma, colon and ovarian cancer [42-45] and in animal models of lung cancer, pancreatic cancer and glioblastoma [46].

Currently, clinical trials with Salirasib have already been completed for pancreatic cancer and for non-small cell lung cancer, demonstrating the safe profile of oral Salirasib, with minimal side effects.

Following *in-vitro* treatment, we detected a decrease in cell viability and migration, G1 cell cycle arrest and a reduction in p-ERK, HIF-1α and EWS-FLI-1 protein levels. We studied the effect of Salirasib in 2 *in-vivo* settings: one similar to the clinical setting in which treatment was administered after the establishment of a tumor, and the second simulating microscopic disease in which treatment was administered simultaneously with tumor cells injection. In both settings a significant inhibition of tumor growth was demonstrated in the treated animals. In the disease microscopic setting, Salirasib was even able to prevent the generation of tumor in 2 mice. This observation suggests the potential use of this drug during post-chemotherapy maintenance to eliminate minimal residual disease.

Furthermore, following Salirasib treatment *in-vivo*, a significant reduction in HIF-1α and EWS-FLI-1 proteins was detected.

In summary, our results support the hypothesis that *let-7* is a tumor suppressor that regulates the RAS signaling pathway, also in ES. Moreover, *let-7* negatively regulates HIF-1α and EWS-FLI-1 proteins. We have shown that HIF-1α directly binds and regulates the chimeric transcript EWS-FLI-1 and contributes to the high proliferation rate and migration ability of ES cells. These results imply that HIF-1α might contribute to the high frequency of metastasis and thereby aggressiveness of ES tumors.

The reduction in tumor burden in a mouse model of ES following Salirasib treatment, demonstrates the therapeutic potential of this RAS inhibitor in ES.

Our findings can set the basis for conducting a phase I-II clinical study evaluating the effect of Salirasib in ES patients.

## MATERIALS AND METHODS

### Patients

Primary tumor samples were obtained from 67 ES patients who were admitted to the Rina Zaizov Pediatric Hematology Oncology Department at the Schneider Children's Medical Center of Israel. The local and national Ethics Committees approved the research project. All patients were treated according to the locally established protocol for ES, which is similar to most common protocols for this malignancy, including a combination of intensive chemotherapy based on VACA and IV-VACA protocols (vincristine, actinomycin-D, cyclophosphamide, adriamycin, ifosphamide, etoposide), radiotherapy, and surgery. Median age at diagnosis was 13 (range 0.3-28). Primary sites were: 31 limb, 20 pelvis, 5 chest, 3 multifocal, and 8 others. Thirty four patients (51%) progressed, 9 locally, 16 in the lungs, and 9 in distal bony sites. Median follow up was 64 months (range 7-260). All tissue samples were snap-frozen in liquid nitrogen immediately after surgery and were stored at −80°C. For some patients tumor samples were available and for some only DNA and/or RNA that had previously been extracted. All tumors harbored the EWS-FLI-1 chimeric transcript.

### Cell lines

ES cell lines SK-ES1, SK-N-MC, MHH-ES, RD-ES1 and the breast cancer cell line MCF-7 were obtained from DSMZ Human and Animal Cell Lines Database and cultured according to the DSMZ growth recommendation. A673 was obtained from ATCC and cultured according to ATCC growth recommendation. ES cell lines 5838, SK-NM-CII, IARC-EW2 and TCL9 were a kind gift from Prof. Yossi Shiloh, Tel Aviv University.

### miRNAs

The Sanger data base, miRBase [15] was used for the detection of the miRNAs located on chromosomes 11q and 22q. For the prediction of target genes we used Target scan and TargetRank sites.

### Real-time quantitative reverse transcription PCR (RQ-PCR)

Total RNA was extracted from 57 tumors using miRNeasy Mini Kit (Qiagen, Valincia, CA, USA) for fresh tumors or miRNeasy FFPE kit (Qiagen, Valincia, CA, USA) for paraffin embedded tissue. The expression profiles of *let-7a* and *let-7b* microRNAs were measured by using miScript SYBR Green PCR Kit (Qiagen, Valincia, CA, USA) by RQ-PCR using the LightCycler 480 (Roche, Mannheim, Germany). High or low *let-7* expression levels were defined above or below the first quartile of each miR. All reactions were run in duplicates and RNA input was normalized using the U6 snRNA endogenous control using the delta Ct(Ct let7-Ct U6) method.

### RAS mutation identification

DNA for the mutation analysis was available from 29 primary tumors. PCR was performed for regions flanking codons 12,13 and 61 of each *RAS* gene with specific primers and ReddyMix PCR Master Mix (ABgene Epson, Surrey, UK) according to the manufacturer's protocol. The PCR products were direct sequenced. The primers were 5′ to 3′: K-RAS-F ggt act ggt gga gta ttt ga K-RAS-R gga tca tat tcg tcc aca aa; K-RAS2-F gca ctg taa taa tcc aga ctg K-RAS-R tac aca aag aaa gcc ctc cc; N-RAS1-F aat gga agg tca cac tag ggN-RAS1-R tca cct cta tgg tgg gat ca; N-RAS2-F ata gca ttg cat tcc ctg tg N-RAS2-R gta cct gta gag gtt aat at; H-RAS1-F ttg ccc ttc aga tgg ccc tg H-RAS1-Rcgc tag gct cac ctc tat ag; H-RAS2-F gca gga ttc cta ccg gaa gc H-RAS2-R cac ctg tac tgg tgg atg tc.

### Western blots

Protein was extracted from ES cell lines using CHAPS lysis buffer containing protease and phosphatase inhibitors (Calbiochem, San Diego, CA, USA). The membrane was then probed with the following antibodies: N-RAS (1:1000), pan-RAS (2mg/ml) (Calbiochem, San Diego, CA, USA), ERK (1:500) (Santa Crus Biotechnology, Santa Crus, CA, USA) phospho-ERK (p-ERK) (1:1000),), HIF-1α (1:250) (Cell Signaling, Danvers, MA, USA), FLI-1 (1:200) (Santa Crus Biotechnology, Santa Crus, CA, USA), GLUT1 (1:500), VEGF (1:500) and α-TUBULIN (1:1000) (Sigma, Saint Louis, Missouri, USA). The secondary antibodies were goat anti-mouse and goat anti-rabbit (1:10,000) (Sigma, Saint Louis, Missouri, USA), for each antigen according to the manufacturer's instructions.

### Immunohistochemistry

Immunohistochemistry was performed on fixed paraffin embedded sections of 45 ES tumors at diagnosis using anti-phospho p44/42 MAPK (ERK1/2 1:70) antibody (Cell Signaling, Danver, MA, USA). Sections of 3.5 micrometer were mounted on SuperFrost Plus glass (Menzel-Glazer, Braunschweig, Germany) and processed by an automated immunostainer (VENTANA ES, Ventana Medical System, Tucson, AZ, USA). Immunohistochemistry was performed using a three-step indirect process [16]. p-ERK protein expression was analyzed by scoring the percentage of labeled cells and the intensity as: + = weak, ++ = moderate, or +++ = strong.

### Fraction extraction

The Qproteome: Cell Compartment kit (Qiagen, Valincia, CA, USA) was used for cell fractionation. The fractions were separated into cytosolic, membrane, nuclear and cytoskeletal fractions on Bis-Tris gel (Invitrogen Corporation, Carlsbad, USA) and Western blot analysis was performed.

### Transfections

Five million SK-ES1 cells were seeded in 10cm plates and transfected with 1nM mimics of *let-7a* or *let-7b* and 1nM inhibitors against *let-7a* or *let-7b* (Qiagen, Valincia, CA, USA) using INTERFERin transfection reagent (Polyplus-transfection, New York, USA), according to the manufacturer's protocol. In addition, SK-ES1 cells were transfected with HIF-1α siRNA (Qiagen, Valincia, CA, USA), using buffer A (20mM PIPES PH = 7, 128Mm K-glutamate, 10mM Ca-acetate, 2Mm Mg-acetate all in 1x McCoy's medium). The cells were electroporated with 40μg of HIF-1α siRNA (Capacitance-900μf, Voltage-350V) and plated in 1x McCoy's medium 15% fetal calf serum (FCS).

### Cell proliferation kit (XTT)

SK-ES1 cells were cultivated in a flat 96-well plate. Salirasib was added for 1 hour and the proliferation rate was measured using the XTT reagent solution (Biological Industries Ltd., Kibbutz Beit Haemek, Israel) according to the recommended protocol. In addition, SK-ES1 cells were incubated in 95% air and 5% CO_2_ at 37°C for 24 and 48 hrs. The cells were transfected with HIF-1α siRNA and cell proliferation was detected by XTT. The absorbance of the samples was measured with a spectrophotometer at a wavelength of 450-500 nanometers. In order to measure reference absorbance (to measure non-specific readings), a wavelength of 630-690 nanometer was used.

### Migration assay

Tumor cell migration was measured using the InnoCyte™ Cell Migration Assay (Calbiochem, San Diego, CA, USA). 2.5×10^5^ SK-ES1 cells following silencing of HIF-1α for 48 hrs, 2.5×10^5^ SK-ES1 cells following 5 days of treatment with 100μM Salirasib and untreated SK-ES1 cells (control), were incubated in triplicate wells of the upper chamber and migrated towards the lower chamber, containing medium with and without 30% FCS, for 5 hrs at 37°C. The results were presented as an average of 3 different experiments. Each was run in triplicates and analyzed using a standard fluorescent plate reader at 485/520nm.

### ChIP assay

The protein/DNA interaction of HIF-1α and EWS was analyzed by ChIP-IT™ Express Kit with protein G-coated magnetic beads (Active Motif, Carlsbad, CA,USA), according to the manufacturer's instructions. Following immunoprecipitation, the chromatin was eluted, cross-linking was reversed and the proteins were removed by treatment with Proteinase K. Before adding an antibody directed against HIF-1α, an aliquot of each sample was removed to be used as an input for PCR. The DNA was analyzed by PCR and the samples were run on 2% agarose gel to determine which DNA fragments were bound by the protein of interest.

### MAPK inhibition

To evaluate the effect of MEK inhibitor, ES cells were plated to 80% confluence and the cells were grown in low serum conditions (1%) for 12hrs. Then, the medium was replaced by 1% FBS with or without (control) 50μM MEK inhibitor (U0126) for 5hrs. Cells were stimulated with medium containing 10% FBS and 100ng/ml IGF-1 and cell lysates were then prepared at the indicated times (0-45min). Afterwards, p-ERK and EWS-FLI-1 protein levels were measured by Western blotting and by graphic illustration. Protein expression was calculated relative to expression levels of tubulin.

### Cell cycle analysis by fluorescence-activated cell sorting (FACS)

Floating and adherent MHH-ES1, SK-ES1, SK-N-MC, EW-2 and 5838 ES cells were collected following five days of Salirasib treatment, washed with PBS, and 0.5 to 1 million cells were stained with propidium iodide. DNA content was analyzed by FACSCALIBUR using ModFitLT cell cycle analysis software (Verity Software House Inc., Topsham, ME, USA). The percentage of cells in each cell cycle phase was compared between treated and untreated cells.

### Salirasib treatment *in-vitro*

The RAS inhibitor, Salirasib (a farnesylthiosalicylic acid agent) (A gift from Concordia Pharmaceuticals, Fort Lauderdale, FL, USA) was prepared as previously described [17]. We determined the IC50 value of Salirasib to be 100μM for MHH-ES-1 and SK-ES1 cells. Cells were incubated with or without 100μM Salirasib for 24 and 48 hours and live cells were counted following staining with trypan blue. Cells were incubated with or without 100μM Salirasib for 5 days for cell cycle analysis.

To study the effect of Salirasib on the RAS pathway, cells were grown to 50% confluence. Afterwards they were serum starved for 12 hours, treated for 1 hour with Salirasib and serum re-activated after 5, 10, 20 and 30, minutes. The levels of p-ERK, HIF-1α and EWS-FLI-1 were measured at each time point.

### Salirasib treatment *in-vivo*

Ten million SK-ES1 and A673 cells in 100μl of PBS were injected subcutaneously above the femur to 12-week-old male NOD-SCID mice. Tumors were observed 4-7 days after injection in 100% of the mice (*n* = 113). Each experiment was ended when the tumors reached 20×20mm^2^. All animal experiments were performed under an approved animal study protocol.

After the generation of tumors in all mice, they were randomly divided into a control group (SK-ES-1 *n* = 41 and A673 *n* = 12) and a Salirasib-treated group (SK-ES1 *n* = 38 and A673 *n* = 22). Mice were treated daily with oral 100ng/Kg Salirasib in 0.5% carbosymethylcellulose (CMC) or with vehicle only. The volume of tumors was measured daily until the mice were sacrificed and the tumor volume was calculated using the formula π(length)(width)^2^/6 [18].

### Statistical analysis

Expression levels of *let-7* and p-ERK protein levels were assessed for potential association with clinical parameters, including patients’ age, primary site and metastases at diagnosis. The associations were evaluated using Fisher's Exact test and *p* < 0.05 was considered to be statistically significant. Distribution of progression free survival (PFS) based on *let-7* expression and p-ERK protein levels were estimated by Kaplan-Meier analysis (using log rank). Kaplan-Meier analysis was also performed following stratification by the presence or absence of metastases at diagnosis. Results were considered significant for *p* values less than 0.05. Correlations between *let-7* expression and p-ERK protein levels were performed using 2-tailed *t*-test. In the mouse model, the volume of tumors was compared between treated and non-treated groups by *t*-test.
